# Supplementary material for: Post-recovery COVID-19 and incident heart failure in the National COVID Cohort Collaborative (N3C) study
Source: Nat Commun. 2022 Jul 15;13:4117. doi: 10.1038/s41467-022-31834-y (PMC9284961; doi:10.1038/s41467-022-31834-y)
Supplement: Supplementary file 1 — Supplementary Information [file 41467_2022_31834_MOESM1_ESM.pdf]

SUPPLEMENTAL MATERIAL

SUPPLEMENTAL TABLE 1: Unadjusted hazard ratios of post-discharge incident heart failure comparing patients hospitalized with versus without COVID-19. The National COVID Cohort Collaborative Study

**Subgroup**	**N**	**HR (95% CI)**
Age category		
<65 years	401,260	1.86 (1.74 -1.98)
≥65 years	157,757	1.33 (1.26 - 1.39)
Sex		
Female	305,185	1.84 (1.73 - 1.95)
Male	253,832	1.51 (1.43 - 1.60)
Race/ethnicity		
White	348,387	1.76 (1.68 - 1.85)
Non-White or Hispanic	210,630	1.68 (1.57 - 1.80)
Cardiovascular medications		
No	316,676	1.82 (1.67 - 1.98)
Yes	242,341	1.63 (1.56 - 1.70)

Footnote: Multivariable Cox regression models based on a total of 559,017 unique patients without missing age, race/ethnicity, or sex.

Cardiovascular medications include angiotensin converting enzyme inhibitor, angiotensin II receptor blocker, beta blocker, or statin medication use

Abbreviations: HR = hazard ratio
