# Supplementary material for: Post-recovery COVID-19 and incident heart failure in the National COVID Cohort Collaborative (N3C) study
Source: Nat Commun. 2022 Jul 15;13:4117. doi: 10.1038/s41467-022-31834-y (PMC9284961; doi:10.1038/s41467-022-31834-y)
Supplement: Supplementary file 3 — Reporting Summary [file 41467_2022_31834_MOESM3_ESM.pdf]

nature portfolio

Corresponding author(s): Marat Fudim

Last updated by author(s): Jun 22, 2022

Reporting Summary

Nature Portfolio wishes to improve the reproducibility of the work that we publish. This form provides structure for consistency and transparency in reporting. For further information on Nature Portfolio policies, see our Editorial Policies and the Editorial Policy Checklist.

Statistics

For all statistical analyses, confirm that the following items are present in the figure legend, table legend, main text, or Methods section.

n/a	Confirmed	
☐	☒	The exact sample size (*n*) for each experimental group/condition, given as a discrete number and unit of measurement
☐	☒	A statement on whether measurements were taken from distinct samples or whether the same sample was measured repeatedly
☐	☒	The statistical test(s) used AND whether they are one- or two-sided<br>*Only common tests should be described solely by name; describe more complex techniques in the Methods section.*
☐	☒	A description of all covariates tested
☒	☐	A description of any assumptions or corrections, such as tests of normality and adjustment for multiple comparisons
☐	☒	A full description of the statistical parameters including central tendency (e.g. means) or other basic estimates (e.g. regression coefficient) AND variation (e.g. standard deviation) or associated estimates of uncertainty (e.g. confidence intervals)
☐	☒	For null hypothesis testing, the test statistic (e.g. *F*, *t*, *r*) with confidence intervals, effect sizes, degrees of freedom and *P* value noted<br>*Give P values as exact values whenever suitable.*
☒	☐	For Bayesian analysis, information on the choice of priors and Markov chain Monte Carlo settings
☒	☐	For hierarchical and complex designs, identification of the appropriate level for tests and full reporting of outcomes
☐	☒	Estimates of effect sizes (e.g. Cohen's *d*, Pearson's *r*), indicating how they were calculated

Our web collection on statistics for biologists contains articles on many of the points above.

Software and code

Policy information about availability of computer code

Data collection	This is a secondary analysis of data collected by the N3C study. The authors had no participation in the data collection
Data analysis	All data management and statistical analyses were conducted and documented in the secure N3C Enclave programming environment, using the programming tools made available in the N3C Enclave (Spark SQL version 3.0.2 or R version 3.5.1)

For manuscripts utilizing custom algorithms or software that are central to the research but not yet described in published literature, software must be made available to editors and reviewers. We strongly encourage code deposition in a community repository (e.g. GitHub). See the Nature Portfolio guidelines for submitting code & software for further information.

Data

Policy information about availability of data

All manuscripts must include a data availability statement. This statement should provide the following information, where applicable:

- Accession codes, unique identifiers, or web links for publicly available datasets
- A description of any restrictions on data availability
- For clinical datasets or third party data, please ensure that the statement adheres to our policy

The data that support the findings of this study may be requested from NCATS (covid.cd2h.org) but restrictions apply. Access to data requires N3C onboarding and following the regulatory and training requirements that all N3C users must abide by.

Human research participants

Policy information about studies involving human research participants and Sex and Gender in Research.

Reporting on sex and gender	Sex was abstracted from the medical record. There were 321,343 females, 265,831 males, and 156 (0.3%) with missing sex information. Study results are presented both in the aggregate and with sex stratification. The data source for our analysis is the N3C Study, which is a enclave of harmonized electronic health records. The N3C study did not recruit study participants and did not require participant consent.
Population characteristics	See below ("Behavioural & social sciences study design"
Recruitment	Collaborating institutions transferred electronic health records to the N3C study. There was no patient recruitment
Ethics oversight	All N3C activities were approved by a central Institutional Review Board at Johns Hopkins University (Reliance Protocol IRB00249128)

Note that full information on the approval of the study protocol must also be provided in the manuscript.

Field-specific reporting

Please select the one below that is the best fit for your research. If you are not sure, read the appropriate sections before making your selection.

☐ Life sciences ☒ Behavioural & social sciences ☐ Ecological, evolutionary & environmental sciences

For a reference copy of the document with all sections, see nature.com/documents/nr-reporting-summary-flat.pdf

Behavioural & social sciences study design

All studies must disclose on these points even when the disclosure is negative.

Study description	Quantitative analysis of data sourced from the National COVID Cohort Collaborative (N3C, covid.cd2h.org), a secure enclave of observational electronic health records
Research sample	587,330 hospitalized patients who were discharged alive, with no previous diagnosis of heart failure. Of these, 257,075 (49% male, 61% White, mean age 51 years) had COVID-19 infection at the time of hospitalization and 330,255 (42% male, 69% White, mean age 42 years) had no COVID-19 infection, either prior to, during, or after the hospitalization. Data sourced from an existing dataset: the National COVID Cohort Collaborative (N3C, covid.cd2h.org). The N3C study data was chosen for our investigation because the N3C is a large, nationally representative sample of patients with and without COVID-19 infection.
Sampling strategy	This is a secondary analysis of electronic health records collected by the N3C study. Our sample size of patients with COVID-19 infection was based upon availability in the N3C study. We included all patients hospitalized with COVID-19 infection in the N3C study that met our inclusion criteria. The sample of patients without COVID-19 infection was chosen from a random sample (15% of the available patients hospitalized without COVID-19 infection in the N3C study). A 15% random sample yielded an approximate a 1:1 sample of patients with vs. without COVID-19 hospitalization. We chose a 15% random sample rather than the entire sample of patients without COVID-19 infection, because computer processing times were severely limited by the very large data.
Data collection	Data collection was performed by the N3C study. We conducted a secondary analysis of these data, but had no part in the data collection. The data that were collected consisted of electronic health records. There were no study participants and no randomized interventions. The N3C is an observational study. Over 60 collaborating institutions transfered electronic health record data to the N3C enclave, under a Johns Hopkins University Reliance Protocol (IRB00249128) or individual site agreements with the US National Institutes of Health (NIH).
Timing	March 1, 2020 – March 31, 2022
Data exclusions	All exclusions were pre-established. We investigated whether post-recovery COVID-19 is associated with incident heart failure. "Incident" heart failure means a new diagnosis of heart failure in someone who has not had a previous heart failure diagnosis. For this reason, we excluded any patients with history of heart failure diagnosed either before or during the index hospitalization. Because we were interested in heart failure associated with post-recovery COVID-19, the included patients were required to recover from COVID-19. For this reason, we excluded any patients who died during the hospitalization. As a result our study population was limited to patients who were discharged alive from the index hospitalization.
Non-participation	Our analysis was based on electronic health records. No participants were involved in the study.
Randomization	We analyzed observational data collected by the N3C study, to investigate whether COVID-19 hospitalization is associated with incident heart failure. It is not ethical nor feasible to randomize patients to have vs. not have COVID-19 hospitalization.

Reporting for specific materials, systems and methods

We require information from authors about some types of materials, experimental systems and methods used in many studies. Here, indicate whether each material, system or method listed is relevant to your study. If you are not sure if a list item applies to your research, read the appropriate section before selecting a response.

Materials & experimental systems

n/a	Involved in the study
☒	☐ Antibodies
☒	☐ Eukaryotic cell lines
☒	☐ Palaeontology and archaeology
☒	☐ Animals and other organisms
☒	☐ Clinical data
☒	☐ Dual use research of concern

Methods

n/a	Involved in the study
☒	☐ ChIP-seq
☒	☐ Flow cytometry
☒	☐ MRI-based neuroimaging
